# E-vapor aerosols do not compromise bone integrity relative to cigarette smoke after 6-month inhalation in an ApoE^–/–^ mouse model

**DOI:** 10.1007/s00204-020-02769-4

**Published:** 2020-05-14

**Authors:** Marie K. Reumann, Jenny Schaefer, Bjoern Titz, Romina H. Aspera-Werz, Ee Tsin Wong, Justyna Szostak, Victor Häussling, Sabrina Ehnert, Patrice Leroy, Wei Teck Tan, Arkadiusz Kuczaj, Christof Audretsch, Fabian Springer, Andreas Badke, Peter Augat, Leticia Quentanilla-Fend, Manuela Martella, K. Monica Lee, Manuel C. Peitsch, Julia Hoeng, Andreas K. Nussler

**Affiliations:** 1 Siegfried Weller Research Institute, BG Unfallklinik, Eberhard Karls University Tuebingen, Schnarrenbergstr. 95, 72076 Tuebingen, Germany; 2 Department of Trauma and Reconstructive Surgery, BG Unfallklinik, Eberhard Karls University, Tuebingen, Germany; 3 PMI R&D, Phillip Morris Products S.A, Quai Jeanrenaud 5, CH-2000 Neuchatel, Switzerland; 4 PMI R&D, Phillip Morris International Research Laboratories Pte. Ltd, Science Park II, Singapore; 5 Department of Radiology, BG Unfallklinik Tuebingen and University Hospital Tuebingen, Tuebingen, Germany; 6 Institute for Biomechanics, BG Unfallklinik Murnau, Germany & Paracelsus Medical University, Salzburg, Austria; 7 Department of Pathology, Eberhard Karls University, Tuebingen, Germany; 8 Altria Client Services LLC, 6601 West Broad Street, Richmond, VA USA

**Keywords:** E-vapor aerosol, Cigarette smoking, ApoE^–/–^ mouse model, Bone structure, Bone biomechanical properties

## Abstract

Cigarette smoke (CS) exposure is one of the leading risk factors for human health. Nicotine-containing inhalable products, such as e-cigarettes, can effectively support tobacco harm reduction approaches. However, there are limited comparative data on the effects of the aerosols generated from electronic vapor products (e-vapor) and CS on bone. Here, we report the effects of e-vapor aerosols and CS on bone morphology, structure, and strength in a 6-month inhalation study. Eight-week-old ApoE^–/–^ mice were exposed to aerosols from three different e-vapor formulations—CARRIER (propylene glycol and vegetable glycerol), BASE (CARRIER and nicotine), TEST (BASE and flavor)—to CS from 3R4F reference cigarettes at matched nicotine concentrations (35 µg/L) or to fresh air (Sham) (*N* = 10 per group). Tibiae were analyzed for bone morphology by µCT imaging, biomechanics by three-point bending, and by histological analysis. CS inhalation caused a significant decrease in cortical and total bone volume fraction and bone density relative to e-vapor aerosols. Additionally, CS exposure caused a decrease in ultimate load and stiffness. In contrast, bone structural and biomechanical parameters were not significantly affected by e-vapor aerosol or Sham exposure. At the dissection time point, there was no significant difference in body weight or tibia bone weight or length among the groups. Histological findings revealed microcracks in cortical bone areas among all exposed groups compared to Sham control. In conclusion, because of the bone-preserving effect of e-vapor aerosols relative to CS exposure, e-vapor products could potentially constitute less harmful alternatives to cigarettes in situations in which bone health is of importance.

## Introduction

Cigarette smoke (CS) exposure is one of the leading preventable risk factors affecting human health (Samet 2013). Cigarette smoking has a range of health impacts including local oral/pharyngeal diseases, chronic obstructive pulmonary disease (COPD), cardiovascular disease, systemic dysfunctions, cellular impairment, and cancer (Brandsma et al. 2019; Huxley and Woodward 2011; Rivera 2015). While smoking cessation is clearly the most effective measure to reduce the harm from smoking-related diseases (Godtfredsen et al. 2008), switching to less harmful products can be an alternative for smokers who otherwise would continue to smoke. In recent years, increasing awareness has led to the development of potentially less harmful alternatives to cigarettes, such as electronic cigarettes (e-cigs), which heat a liquid containing propylene glycol, vegetable glycerin, nicotine, and flavors to generate an aerosol. In comparison to CS, e-vapor product aerosols deliver nicotine with reduced levels of harmful and potentially harmful constituents (HPHC) (Goniewicz et al. 2013). The British National Health Service recognizes that e-cigs are less harmful nicotine delivery products than cigarettes because of the reduced levels of harmful constituents (tar and carbon monoxide) in e-vapor aerosol (NHS 2019).

The effects of CS on bone have been investigated quite extensively in the past, both in vivo and in vitro (Kallala et al. 2013; Ward and Klesges 2001; Wu et al. 2016). Cigarette smokers have a significantly higher risk of fracture than non-smokers, with the highest risk being observed for hip fracture (Kanis et al. 2005). Additionally, Yoon et al. demonstrated that cigarette smoking increases the risk for osteoporotic fractures through alteration of the RANK–RANKL–OPG system (Yoon et al. 2012). Moreover, in murine models, previous studies showed that bone structure and strength were significantly reduced after passive CS exposure by housing rats for (short term) 4 and 8 weeks in a chamber ventilated with CS (Ajiro et al. 2010) as well as after long-term 12-week sidestream CS exposure in a mouse model (Akhter et al. 2005). Supporting these data, El-Zawawy et al. showed reduced callus formation and delayed bone healing in mice in response to CS exposure in a smoking chamber (El-Zawawy et al. 2006). Probing potential molecular mechanisms, in vitro data demonstrated that exposure to cigarette smoke extract leads to an alteration of antioxidative enzymes caused by nicotine and cotinine (Aspera-Werz et al. 2018).

Only within the last few years have the molecular effects of e-vapor aerosols on human health been investigated in detail (Gotts et al. 2019; Polosa et al. 2019). However, the effect of nicotine on osteogenic cells is not yet fully elucidated and might depend on concentration and exposure time. Marinucci et al. (Marinucci et al. 2018) showed that nicotine induced apoptosis in human osteoblasts via increased reactive oxygen species levels. Moreover, SaOS-2 cells exposed to nicotine for 14 days displayed reduced matrix formation (Tanaka et al. 2005). In contrast, Daffner et al. (Daffner et al. 2012) reported a nicotine-induced increase in osteoblast activity in bone marrow stromal cells. Additionally, our own data showed that nicotine and cotinine, when applied at levels found in the serum of smokers, did not alter in vitro osteogenic differentiation (Aspera-Werz et al. 2018). This might suggest that the detrimental effects on bone-forming cells are mediated by other molecular species present in CS.

It can be concluded that devices that administer nicotine (e.g., e-cigs, heat-not-burn tobacco products, and nicotine sprays or patches) could potentially be less harmful alternatives to cigarette smoking and thus help protect bone structure. To our knowledge, there has been no study on the effects of e-vapor aerosol on the musculoskeletal system and specifically on bone in murine models.

Here, we comparatively investigated the effects of e-vapor aerosols and CS in the ApoE^–/–^ mouse model. The ApoE^–/–^ mouse model is especially well suited for concomitantly assessing the effects of CS on the respiratory and cardiovascular systems (Lo Sasso et al. 2016; von Holt et al. 2009). The bone-related results presented here are part of a larger 6-month assessment, in which we also investigated cardiovascular and lung-associated changes due to e-vapor aerosol exposure compared with cigarette smoking in ApoE^–/–^ mice (Szostak et al. 2020). ApoE^–/–^ mice have been studied in the past, and their bone phenotype has also been investigated. Schilling et al. (Schilling et al. 2005) reported that 3- and 8-month-old ApoE^–/–^ mice had greater bone mass than wild-type animals and showed an increased bone formation rate, although bone resorption was not affected. This aspect might enable a more sensitive detection of bone-related effects due to CS and e-vapor aerosol exposure in this mouse model. Additionally, others have previously used this model to investigate the effect of CS on bone strength in female C57BL (ApoE^–/–^) mice (Akhter et al. 2005), showing evidence of a significant reduction in biomechanical properties due to CS exposure.

The aim of this study was to investigate if the effects of e-vapor aerosol exposure on bone structure and strength are less harmful than those of CS exposure and, additionally, to investigate the effect of e-vapor aerosol components (humectants, flavor, and nicotine).

## Materials and methods

This work is part of a comprehensive inhalation toxicology study on e-vapor aerosols compared with CS exposure; the study also includes assessment of systemic, respiratory, and cardiovascular effects in the ApoE^–/–^ mouse model. Here, we summarize the main procedures relevant to the investigation of the effects of exposure on bone in this study; for further details and other endpoints, the reader is referred to the other topical reports on this study (Szostak et al. 2020), as well as to the corresponding data sets on INTERVALS (https://www.intervals.science/studies/#/apoe_p4).

### Animal model

All procedures performed in this study involving animals were in accordance with the ethical standards of the institution or practice at which the study was conducted (a facility accredited by the Association for Assessment and Accreditation of Laboratory Animal Care (AAALAC) and licensed by the Agri-Food & Veterinary Authority of Singapore, with approval from an Institutional Animal Care and Use Committee (IACUC, protocol #15044)) and in compliance with the National Advisory Committee for Laboratory Animal Research Guidelines on the Care and Use of Animals for Scientific Purposes (Naclar 2004). All applicable international, national, and institutional guidelines for the care and use of animals were followed.

Female ApoE^–/–^ mice (B6.129P2-Apoe^tm1/Unc^ N11) bred under specific pathogen-free conditions were obtained from Taconic Biosciences (Rensselaer, NY, USA). The health status of the animals was verified using the health check certificate provided by the breeder. Mice were maintained and exposed under specific hygienic conditions as described previously, with filtered conditioned fresh air at 22 ℃ ± 2 ℃ and 55% ± 15% humidity. Additional details of animal housing, randomization, and acclimatization have been published previously (Boue et al. 2012; Lietz et al. 2013; Phillips et al. 2016a).

### Animal groups and exposure

**Fig. 1:**
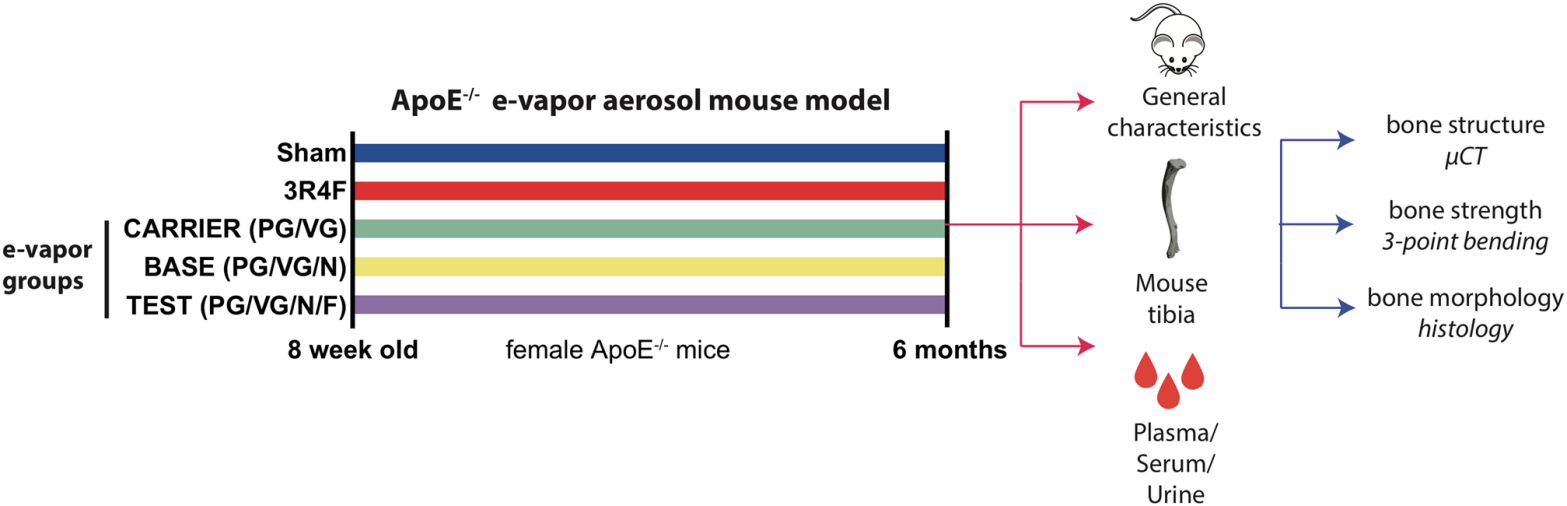
Study design: the study is based on an ApoE^–/–^ e-vapor aerosol mouse model. Female ApoE^–/–^ at the age of 8 weeks were exposed to five treatment regimen: Sham, 3R4F and the three e-vapor groups (CARRIER, BASE and TEST). Duration of treatment lasted for 6 months. When mice were sacrificed, general characteristics (mouse body weight, tibial size and weight after dissection) were analyzed. Systemic analysis of specific parameters (nicotine, cotinine, propylene glycol, total nicotine metabolites, CoHb) was performed from plasma, serum and urine at two time points (~3 months and ~6 months during exposure). Specific bone integrity analysis was performed, including bone structure analysis using µCT, bone strength analysis using three-point bending and bone morphology using histological stainings

At the age of 8 weeks, mice were randomly allocated using the body weights to five exposure groups and subjected for up to 6 months of whole-body exposure (Table 1): Sham (exposure to fresh, conditioned air); diluted mainstream CS from the 3R4F reference cigarette; or three groups of e-vapor aerosol exposure (CARRIER, BASE, and TEST). The CARRIER e-vapor liquid contained the humectants propylene glycol (PG) and vegetable glycerin (VG) alone; BASE contained the humectants and nicotine; and TEST contained the humectants, 4% nicotine, and flavors (Table 2). For the BASE and TEST formulations containing nicotine (4%), mixtures of acids (1%) were added, with the resulting pH of ~8. The BASE and TEST group exposure atmospheres were configured to deliver a nicotine concentration of 35 µg/L (corresponding to the nicotine level of 560 µg/L total particulate matter (TPM) from 3R4F cigarettes). Schematic in Fig. 1 gives an overview of the general study design (Fig. 1).

**Table 1:** Mouse groups treated with various exposure conditions

Group	Composition	Exposure
Sham	Sham	Fresh conditioned air
3R4F	3R4F	Cigarette smoke (CS) from reference cigarette 3R4F
CARRIER	PG/VG	Humectants (propylene glycol (PG) and vegetable glycerin (VG)) aerosol
BASE	PG/VG/N	Humectants (PG/VG) and nicotine (N) aerosol
TEST	PG/VG/N/F	Humectants (PG/VG), nicotine (N), and flavors (F) aerosol

**Table 2:** Mass compositions of tested e-cigarette liquids

Component	CARRIER (g/1000g)	TEST (g/1000g)	BASE (g/1000g)
Propylene glycol (PG)	255.00	240.00	238.91
Vegetable glycerin (VG)	595.00	560.01	559.90
Water	150.00	150.00	150.00
Nicotine	0.00	40.00	40.00
Benzoic acid	0.00	3.33	3.33
Lactic acid	0.00	3.33	3.33
Acetic acid	0.00	3.33	3.33
Flavor blend	0.00	0.00	1.20
Sum	1000.00	1000.00	1000.00

Components of flavor blend are proprietary

Mice were exposed for 3 h per day, 5 days per week, for up to 6 months. Intermittent exposure to fresh filtered air for 30 min after the first hour of exposure and for 60 min after the second hour of exposure was provided to avoid accumulation of excessive carboxyhemoglobin (COHb) in the 3R4F group.

3R4F reference cigarettes were purchased from the University of Kentucky (College of Agriculture 2019). Mainstream CS from 3R4F cigarettes was generated on 30-port rotary smoking machines in accordance with the Health Canada intense smoking protocol (Health_Canada (1999)), which is based on ISO standard 3308 (ISO3308 1991.): 55-mL puff volume, one puff per 30 s, and 100% blockage of ventilation holes (Government 2000); the 3R4F puff count was 10–11 per cigarette (average, 10.4 ± 0.3). For whole-body exposure, the concentration of 3R4F CS was 562 ± 65 (mean ± standard deviation) µg TPM per liter (600 µg /L target concentration) and 35.2 ± 4.8 (mean ± standard deviation) µg nicotine/L.

E-vapor aerosols for the CARRIER, BASE, and TEST groups were prepared by adding each component and diluting to the final mass composition (Table 2). The prepared mix was stored away from light, at a controlled temperature of 2–8 ℃ under uncontrolled humidity conditions. The CARRIER, BASE, and TEST aerosols were generated using a capillary aerosol generator developed by Philip Morris International Inc. and further refined by Virginia Commonwealth University (Gupta et al. 2003; Howell and Sweeney 1998). This aerosol generator was previously shown to deliver aerosols in a consistent manner and at similar particle size distribution and concentrations as a prototype e-cig device (Werley et al. 2016). The temperature of the capillary aerosol generator was set at 250–275 °C to match the temperature of the heated coil during puffing of the e-cig device (Geiss et al. 2016). The generator was fitted with a diffuser and compressed air to prevent aerosol backflow. Condensation aerosol was created when the output from the generator was diluted with air at ambient temperature. A portion of the formed aerosol from the generator was further diluted with filtered air to achieve the target concentrations in the test atmosphere and delivered via glass tubing to the exposure chamber.

For whole-body exposure, the CARRIER aerosol contained PG at a concentration of 179.4 ± 21.3 µg/L and VG at a concentration of 576.9 ± 65.6 µg/L (without nicotine). The BASE aerosol was nicotine-matched to 3R4F and PG/VG-matched to the CARRIER (35.5 ± 4.9 µg nicotine/L, 171.3 ± 16.7 µg PG/L, and 543.5 ± 68.4 µg VG/L); the TEST aerosol was also nicotine-matched to 3R4F and PG/VG-matched to the CARRIER (35.7 ± 5.6 µg nicotine/L, 173.0 ± 19.4 µg PG/L, and 546.2 ± 74.2 µg VG/L).

### Analysis of biomarkers of exposure

Blood COHb concentrations were determined as described previously (Phillips et al. 2016a; Phillips et al. 2015). Blood was collected from the facial vein under anesthesia within 15 min after exposure (at months 2 and 5). For plasma collection, blood was placed on ice after collection and processed. Aliquoted plasma was transferred to storage at ≤  − 70 °C. Plasma PG, nicotine, and cotinine concentrations were measured by ABF GmbH (Planegg, Germany). Urine was collected during exposure and for approximately 18 h post exposure in individual metabolic cages. Urine collected during exposure, urine from the 18 h overnight collection, and water collected during rinsing of the cage (with approximately 100 µL of water) were pooled per animal, aliquoted, and stored at ≤ − 70 °C. Nicotine metabolites (trans-3′-hydroxycotinine, norcotinine, cotinine, nicotine-*N*′-oxide, and nornicotine) in urine were analyzed by LC-MS/MS after 1,3-diethyl-2-thiobarbituric acid derivatization at ABF.

### Tissue preparation

The bone phenotype of female ApoE^–/–^ mice exposed to various smoke/aerosol conditions was determined by micro-computed tomography (µCT), biomechanical testing by three-point bending, and histological analysis of intact tibiae. On the scheduled necropsy date and approximately 16–24 h after the last exposure, mice were anesthetized with 100 mg/kg pentobarbital before exsanguination and perfusion with 0.9% saline. Both tibiae of each animal were harvested by disarticulation of the hip joint to separate the lower limb from the mouse torso as described before (Reumann et al. 2011a, b). The patellar tendon was cut horizontally, and the tibia was separated from the femur. Subsequently, all surrounding muscle was removed from both bones. The fibula was removed along with the soft tissue. As some tibiae got damaged during preparation, only intact bones were further analyzed and served for general characteristics (Sham: *N* = 10; 3R4F: *N* = 16; CARRIER: *N* = 13; BASE: *N* = 11; TEST: *N* = 14). For bone length analysis, X-ray images were acquired using an X-ray Bucky table (BuckyDiagnost CS Optimus, Philips, Hamburg, Germany) and standard cassettes (IP Cassette Type CC, FCR standard cassette 18x24cm, Fujifilm, Germany). Settings for the index finger (50 kV; 2.5 mAs; free exposure with small focal spot of 0.6; and a focus-detector distance of 1.05 m) were used to achieve high spatial resolution for the small bones. X-ray images were analyzed using an electronic PACS system (IMPAX 6.5.5.1033 Version 2014, AGFA HealthCare N.V., Mortsel, Belgium). Bone weight analysis was performed for all intact tibiae using the Kern ABJ scale (Kern & Sohn GmbH, Balingen, Germany).

### Characterization of bone architecture

The overall 3D morphology of total bone compartment of the whole intact right tibiae (*N* = 7/group) was assessed using a Scanco µCT 80 system (Scanco Medical, Bassersdorf, Switzerland). Additionally, a detailed analysis of the midshaft cortical area was performed. The midshaft cortical region of interest was defined in the middle area of the long bone. Each bone was measured in length and the exact midpoint was used as landmark. From there, a total of 50 sections, 25 sections distal and proximal from landmark, were defined for further midshaft analysis for each bone. All bones were scanned in 4% formalin. Parameters of 20 µm voxel size, 70 KVp, a 200 ms exposure and one frame per view were used for the scans. The Scanco µCT software (µCT evaluation program V6.0, Scanco Medical, Switzerland) was used for 3D reconstruction, evaluation and viewing of images. After 3D reconstruction, the volumes of interest were segmented and analyzed for whole bone and for midshaft cortical bone. Directly measured bone volume fraction (BV/TV), mean/density TV and mean/density were calculated for all whole bone (Table 3). BV/TV, total area (TA), bone area (BA) and bone total area ratio (BA/TA) of the cortex, polar (pMOI), maximum (Imax) and minimum (Imin) moments of inertia were calculated for the midshaft cortical areas of the bones (Table 4).

**Table 3:** Parameters for μCT analysis for whole bones.

Parameters for whole bone evaluation		Unit
TV	Total volume	(mm^3^)
BV	Bone volume	(mm^3^)
BV/TV	Bone volume fraction	(ISO3308 1991)
Mean/ density of TV (apparent)	Hydroxyapatite per ccm in total volume	(mm HA/ccm)
Mean/ density of BV (material)	Hydroxyapatite per ccm in bone volume	(mm HA/ccm)

**Table 4:** Parameters for μCT analysis for midshaft cortical bone area.

Parameters for midshaft cortical bone evaluation		
pMOI (polar MOI)	Polar moment of inertia	(mm^4^)
*I*_max_	Maximum polar moment of inertia	(mm^4^)
*I*_min_	Minimum polar moment of inertia	(mm^4^)
BA	Bone area	(mm^2^)
TA	Total area	(mm^2^)
BA/TA	Bone area fraction	(ISO3308 1991)

### Characterization of bone strength

The mechanical properties of intact left tibiae were evaluated by a three-point bending test (Sham: *N* = 6; 3R4F: *N* = 8; CARRIER: *N* = 6;  TEST: *N* = 8; BASE: *N* = 7). The tests were conducted at room temperature on a precision load frame (Zwicki Z2.5 TN, Zwick Roell, Ulm, Germany). The tibiae were tested with their posterior side loaded in compression and the anterior side in tension. The anterior surface was placed on the two lower supports, which were set 10 mm (60% of mean tibial length) apart for all tibiae of all groups. Load was applied at 0.025 mm/s, with a preload of 1 N, until failure. Structural mechanical properties dependent on geometry were measured using the testXpert II V3.3 program (Zwick Roell, Ulm, Germany). Parameters included ultimate load (N), stiffness (N/mm), work to fracture (Nmm), and post-yield displacement (PYD; mm). The setup and parameters were based on those reported by Jepsen et al. (Jepsen et al. 2015).

### Characterization of bone morphology

All dissected tibia samples (Sham: *N* = 4; 3R4F: *N* = 2; CARRIER: *N* = 3; BASE: *N* = 3; TEST: *N* = 3) were fixed in 4% formalin at room temperature for 24 h. All samples were embedded in paraffin after decalcification in ethylenediaminetetraacetate (10% EDTA, pH 7.4) for 48 h at 37 °C. For each sample, serial longitudinal sections (2.5 µm thickness) were prepared, deparaffinized in xylene, and rehydrated in an ethanol gradient. The sections were then stained with hematoxylin and eosin (H&E), Alcian blue, Masson trichrome stain, and Van Gieson stain using standard procedures. Digital bright field images from slides were captured using a Mirax Scan or Axio 2 imaging system (Carl Zeiss MicroImaging GmbH, Jena, Germany).

### Statistical analysis

Statistical analysis for the bone-related endpoints was performed using GraphPad Prism 5.0 (La Jolla, CA, USA). Differences between the groups were evaluated by analysis of variance, the Kruskal–Wallis *H*-Test followed by Dunn’s multiple comparison test. Significance was defined as *p* < 0.05. All data are shown as box-and-whisker (Tukey) plots, indicating the median and interquartile range (IQR). Whiskers show the 1.5 IQR. Individually plotted values are outliners beyond the 1.5 IQR.

More details on the exposure characterization data and statistical analysis are available in Szostak et al. (Szostak et al. 2020). Data are expressed as mean ± standard error of the mean. Pairwise comparisons between groups were performed, and unadjusted *p *values are reported. For continuous variables, if the data of the two groups being compared did not exhibit strong deviation from the normal distribution (as assessed by a Shapiro-Wilk test at the 5% level on the standardized residuals of both groups), a two-sample *t*-test accounting for variance heterogeneity was performed. Otherwise, an exact Mann–Whitney–Wilcoxon two-sample test was used (Monte Carlo estimates of the exact *p* values were used). Results were considered significantly different for a specific comparison if *p*<0.05.

## Results

### Exposure characterization

To characterize exposure and uptake, nicotine, cotinine, and PG were measured in plasma and nicotine metabolites were measured in urine; in addition, COHb —as a marker of carbon monoxide exposure—was measured in blood (Tables 5, 6). The nicotine-containing smoke/aerosol exposure groups (3R4F, BASE and TEST) showed comparable systemic levels of nicotine, cotinine, and nicotine metabolites. Exposure to PG-containing aerosols (CARRIER, BASE, and TEST) also yielded similar plasma levels of PG. 3R4F CS exposure resulted in an increase in COHb levels. As mentioned before, the current investigation on the effects of exposure on bone was a part of a larger systems toxicology study. For further details on exposure characterization and other endpoints, the reader is referred to the other topical reports on this study (Szostak et al. 2020).

**Table 5:** Exposure characterization at time point 1

	Time point 1				
	Sham	3R4F	CARRIER	BASE	TEST
Nicotine ng/Ml (plasma)	0.00 ± 0.00	236.41 ± 87.48 *	0.00 ± 0.00	132.32 ± 39.61 *	115.01 ± 23.04 *
Cotinine ng/mL (plasma)	0.00 ± 0.00	262 ± 37 *	0.00 ± 0.00	270 ± 37 *	287 ± 42 *
Propylene glycol µg/mL (plasma)	0.1 ± 0.01	0.17 ± 0.02 *	3.31 ± 0.85 *	4.37 ± 1.59 *#	3.18 ± 0.76 *#
Total nicotine metabolites µmol (urine)	0.0004 ± 0.00007	0.67 ± 0.05 *	0.00035 ± 0.0001	0.72 ± 0.07*	0.81 ± 0.15 *
COHb % (blood)	2.9 ± 0.03	31.9 ± 2.11 *	3.0 ± 0.05	2.9 ± 0.05 #	2.9 ± 0.03 #

Mean ± SEM; * = versus Sham *p* < 0.05; # = BASE or TEST versus 3R4F *p* < 0.05; *N* = 8 / group

Measurements performed at time point 1: plasma measurements: day 22–26; urine measurements: day 30–37; blood measurements: day 64–71

**Table 6:** Exposure characterization at time point 2.

	Time point 2				
	Sham	3R4F	CARRIER	BASE	TEST
Nicotine ng/mL (plasma)	0.74 ± 0.55	99.39 ± 17.18 *	0.30 ± 0.30	142.96 ± 53.19 *	83.21 ± 14.31 *
Cotinine ng/mL (plasma)	0.00 ± 0.00	210 ± 23 *	0.00 ± 0.00	295 ± 28 *#	288 ± 37 *
Propylene glycol µg/mL (plasma)	0.17 ± 0.04	0.12 ± 0.02	2.13 ± 0.44 *	2.51 ± 1.05 *#	1.42 ± 0.24 *#
Total nicotine metabolites µmol (urine)	0.0002 ± 0.00003	0.67 ± 0.06 *	0.0002 ± 0.00005	0.8 ± 0.07 *	0.8 ± 0.04 *
COHb % (blood)	3.1 ± 0.05	37.2 ± 2 *	3.0 ± 0.05	3.0 ± 0.03 #	3.2 ± 0.1 #

Mean ± SEM; * = versus Sham *p* < 0.05; # = BASE or TEST versus 3R4F *p* < 0.05; *N* = 8 / group

Measurements performed at time point 2: plasma measurements: day 106–110; urine measurements: day 121–131; blood measurements: day 148–155

### E-vapor aerosol and CS exposure did not alter the general characteristics of ApoE^–/–^ mice

**Fig. 2:**
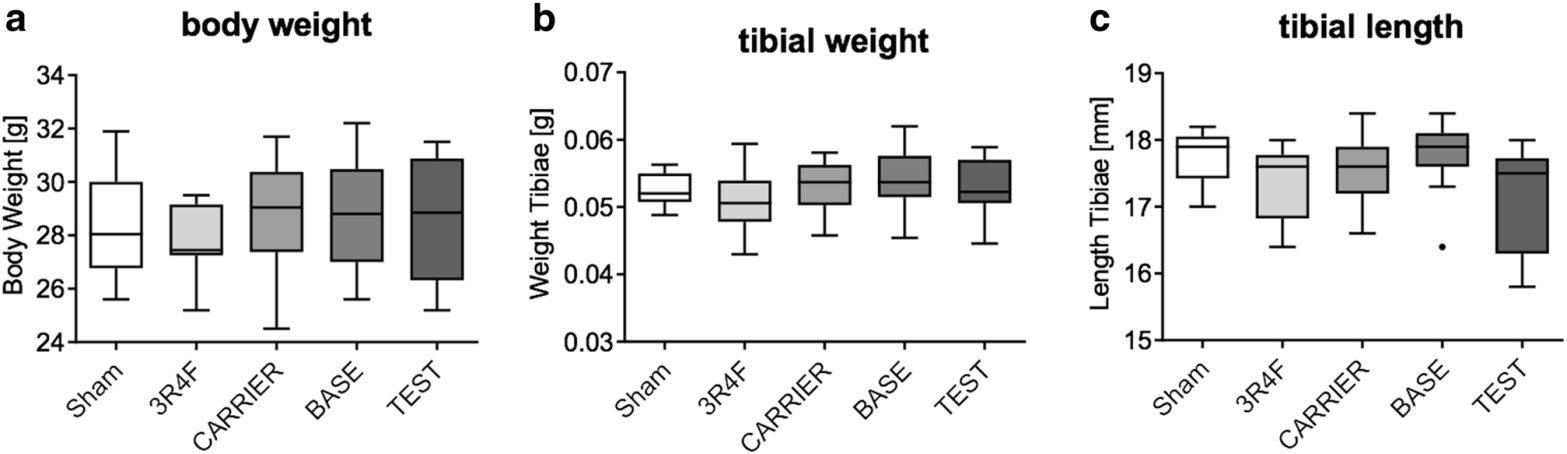
General bone characteristics: Mouse **a** total body weight, **b** tibial weight, and **c** tibial length were measured for ten animals per groups and ≥10 tibiae per group. There was no significant difference in these baseline characteristics among the groups

The general characteristics, body weight, and tibia bone weight and length of ApoE^–/–^ mice were measured to evaluate the effect of e-vapor aerosol inhalation compared with CS. Data analysis showed no significant difference in mouse body weight, tibial weight, or tibial length among the groups (Fig. 2a–c). However, note that, consistent with previous studies (Phillips et al. 2016a; Phillips et al. 2015), the 3R4F group demonstrated significantly lower body weights compared with Sham prior to the 6-month time point of this investigation on the bone-related effects (Szostak et al. 2020).

### The effects of e-vapor aerosol exposure on bone structure were less severe than those of 3R4F CS exposure

**Fig. 3:**
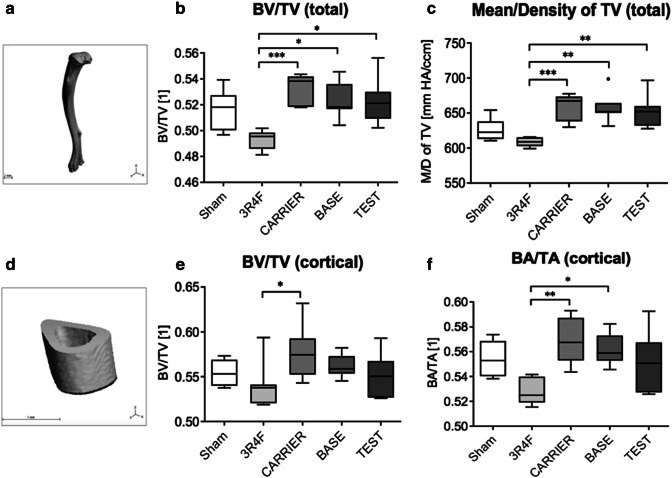
Bone structure analysis: Tibiae were analyzed by µCT for (**a**–**c**) total bone and (**d**–**f**) cortical bone architecture. **a** An overview of a total tibia scanned for total bone analysis. **b** Total bone analysis revealed a significant decrease in BV/TV in mice exposed to 3R4F CS relative to all other e-vapor aerosol groups. **c** 3R4F CS exposure caused a decrease in hydroxyapatite content in the total bone (mean/density of total bone volume). There were no significant differences among the e-vapor aerosol groups. **d** An overview of the midshaft cortical area of a tibia scanned for total bone analysis. **e** Cortical bone volume fraction (cortical BV/TV) was significantly decreased in the 3R4F CS group relative to the CARRIER group. **f** The fraction of cortical bone area (total BA/TA) was significantly reduced in the 3R4F CS group relative to the CARRIER and BASE groups. Results represent mean and 1.5 IQR. Statistical significance was determined by the Kruskal–Wallis test, followed by Dunn’s multiple comparison test. Significance was established as *p < 0.05 and **p < 0.01. *N* = 7/group

Total bone analysis (Fig. 3a) revealed a significantly reduced bone volume fraction (BV/TV) in mice exposed to 3R4F CS relative to the mice in all other e-vapor aerosol groups (CARRIER, BASE, and TEST) (Fig. 3b). In addition, relative to e-vapor aerosol (CARRIER, BASE, and TEST) exposure, 3R4F CS exposure caused a decrease in hydroxyapatite content in total bone (total bone volume density) (Fig. 3c). There was no significant difference in this regard between the e-vapor aerosol and Sham groups or among the three e-vapor aerosol groups (CARRIER, BASE, and TEST). Data on the defined midshaft cortical area (Fig. 3d) revealed significantly decreased cortical bone volume fraction (cortical BV/TV) in the 3R4F group relative to the CARRIER group (Fig. 3e). Similarly, cortical bone area fraction (cortical BA/TA) was significantly reduced in the 3R4F group relative to the CARRIER and BASE groups (Fig. 3f). There was no statistically significant difference in this regard between the sham and 3R4F groups. None of the other µCT parameters showed statistically significant differences among the study groups (Table 7).

**Table 7:** Detailed results of bone structure analysis using μCT

	μCT analysis				
	Sham	3R4F	CARRIER	BASE	TEST
Polar moment of inertia (pMOI)	0.2 ± 0.02	0.19 ± 0.01	0.19 ± 0.01	0.18 ± 0.01	0.2 ± 0.01
Maximum polar moment of inertia (*I*_max_)	0.12 ± 0.01	0.11 ± 0.01	0.12 ± 0.01	0.10 ± 0.01	0.12 ± 0.01
Minimum polar moment of inertia (*I*_min_)	0.08±0.004	0.08±0.01	0.08±0.003	0.08±0.002	0.08±0.003
Maximum loading resistence (*I*_max_/C_max_)	0.16±0.008	0.15±0.007	0.16±0.005	0.15±0.005	0.16±0.005
Minimum loading resistence (*I*_min_/C_min_)	0.13±0.01	0.12±0.006	0.12±0.004	0.12±0.002	0.13±0.003
Bone area (BA)	0.68±0.02	0.64±0.02	0.68±0.02	0.66±0.01	0.68±0.01
Total area (TA)	1.21±0.04	1.21±0.04	1.19±0.02	1.17±0.02	1.23±0.02
Bone area fraction (BA/TA)	0.56±0.01	0.53±0.004	0.57±0.01#	0.56±0.01#	0.55±0.01
Total volume (cortical) (TV (cortical))	1.22±0.04	1.22±0.04	1.19±0.02	1.17±0.02	1.23±0.02
Bone volume (cortical) (BV (cortical))	0.68±0.02	0.65±0.02	0.69±0.01	0.66±0.01	0.68±0.01
Bone volume fraction (cortical) (BV/TV (cortical))	0.56 ± 0.01	0.54 ± 0.01	0.58 ± 0.01#	0.56 ± 0.01	0.55 ± 0.01
Hydroxyapatite content of total volume (cortical) (mean/density of TV (cortical))	696.0 ± 11.31	659.1 ±1 3.83	718.0 ± 11.18	719.1 ± 12.34	711.9 ± 14.70
Hydroxyapatite content of bone volume (cortical) (mean/density of BV (cortical))	1302±9.12	1282±6.59	1296 ± 4.73	1303 ± 4.34	1294 ± 3.61
Total volume (total) (TV (total))	28.35 ± 0.77	28.36 ± 0.27	29.44 ± 0.33	28.48 ± 1.01	28.65 ± 0.61
Bone volume (total) (BV (total))	14.65 ± 0.4	13.99 ± 0.2	15.68 ± 0.14#	14.89 ± 0.55	14.96 ± 0.39
Bone volume fraction (total) (BV/TV (total))	0.52 ± 0.006	0.49 ± 0.003	0.53 ± 0.004#	0.52 ± 0.005#	0.52 ± 0.007#
Hydroxyapatite content of Total Volume (total) (mean/density of TV (total))	627.3 ± 5.96	608.5 ± 2.29	659.6 ± 6.99#	657.4 ± 7.81#	654.1 ± 8.53#
Hydroxyapatite content of bone volume (mean/density of BV (total))	1226 ± 4.33	1213 ± 3.11	1220 ± 3.76	1225 ± 5.5	1223 ± 4.83

Mean ± SEM; * = versus Sham *p* < 0.05; # = versus 3R4F *p* < 0.05; *N* = 7/group.

### E-vapor aerosol exposure exerted smaller effects on bone biomechanical stability than 3R4F CS exposure

**Fig. 4:**
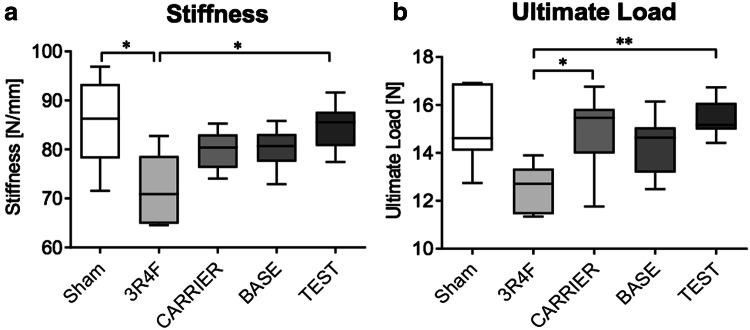
Biomechanical stability analysis of tibiae by the three-point bending test: **a** exposure to 3R4F CS caused a significant decrease in stiffness relative to Sham and TEST aerosol exposure. **b** Ultimate load was significantly reduced in the 3R4F CS group relative to the CARRIER and TEST groups. Results represent mean and 1.5 IQR. Statistical significance was determined by the Kruskal–Wallis test, followed by Dunn’s multiple comparison test. Significance was established as *p < 0.05 and **p < 0.01. Sham: *N* = 6; 3R4F: *N* = 8; CARRIER: *N* = 6; TEST: *N* = 8; BASE: N = 7

The 3R4F CS group showed significantly reduced stiffness relative to the Sham and TEST groups and significantly reduced ultimate load relative to the CARRIER and TEST groups, respectively (Fig. 4a and b). There were no significant differences in these parameters among the e-vapor aerosol exposure groups (CARRIER, BASE, and TEST). Additionally, comparison of the e-vapor aerosol exposure groups to the Sham group did not reveal significant differences, with all groups showing similar biomechanical properties. Post-yield displacement was not affected by any exposure regimen (Table 8). Work to fracture was significantly reduced by 3R4F CS exposure relative to TEST exposure (Table 8).

**Table 8:** Detailed results of biomechanical analysis using three-point bending test

	Biomechanical analysis				
	Sham	3R4F	CARRIER	BASE	TEST
Ultimate load	15.06 ± 0.65	12.52 ± 0.35	14.95 ± 0.69#	14.36± 0.42	15.39 ± 0.29#
Stiffness	85.60 ± 3.71#	71.99 ± 2.53*	79.89 ± 1.61	80.21± 1.43	84.60 ± 1.85#
Work to fracture	4.42 ± 0.23	4.05 ± 0.36	5.15 ± 0.34	4.02 ± 0.32&	5.42 ± 0.22#
Post-yield displacement (PYD)	0.37 ± 0.02	0.5 ± 0.08	0.6 ± 0.08	0.39 ± 0.05	0.61 ± 0.07

Mean ± SEM, *versus Sham *p* < 0.05, ^#^versus 3R4F *p* < 0.05, ^&^versus TEST *p* < 0.05, Sham: *N* = 6, 3R4F: *N* = 8, CARRIER: *N* = 6, BASE: *N* = 7, TEST: *N* = 8

### Bone morphology analysis revealed microcracks in cortical bone areas in all groups

**Fig. 5:**
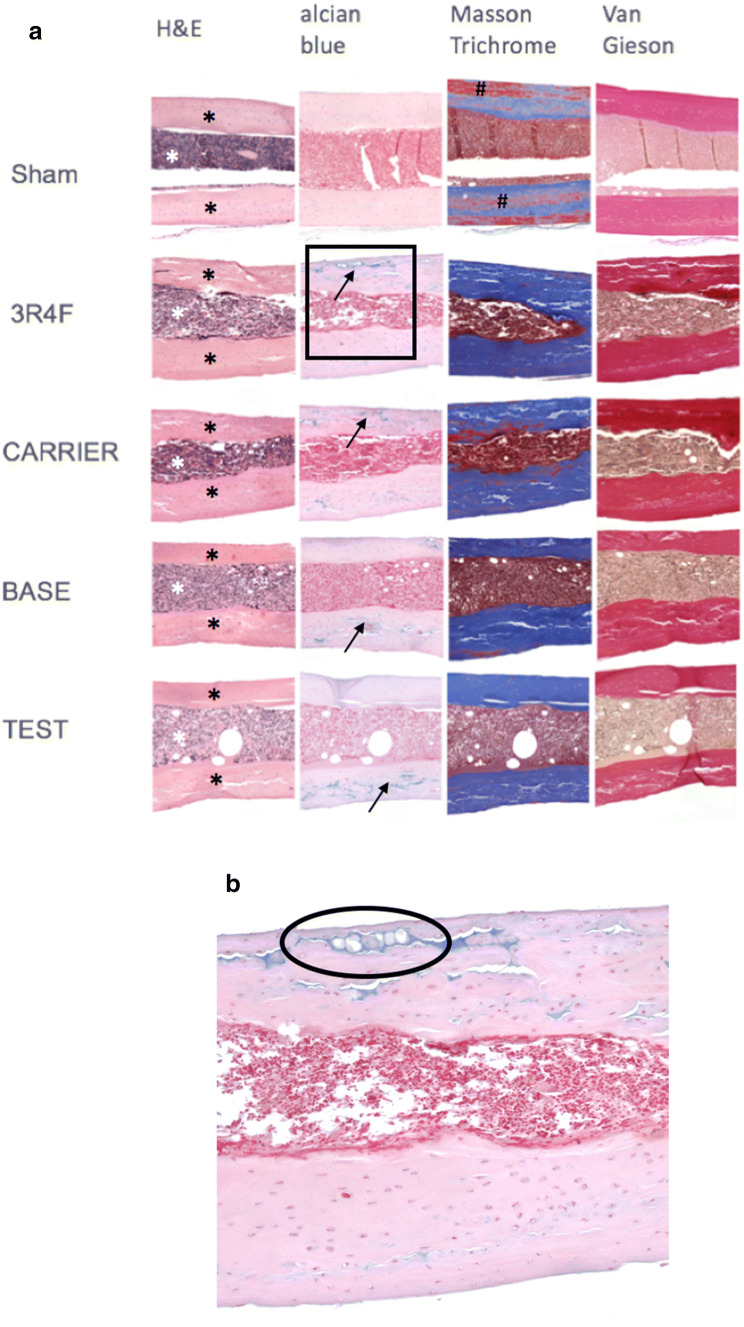
Bone morphology analysis by general histological analysis: **a** all groups were analyzed by histological staining. H&E staining provided a general overview of the cortical tissue (black stars) and cell-enriched intramedullary bone area (white star). Alcian blue staining depicted hypertrophic chondrocytes within cortical bone areas across all treated groups (black arrows). Masson trichrome staining showed red staining in cortical areas, mainly in the sham group (#). Cortical areas consisting of collagen were stained blue; red staining might show a variation in the types of collagen. Van Gieson staining revealed mineralized dark red areas in cortical bone, and mainly intramedullary cell cytoplasm was stained light brown. Additionally, in all treated groups, cortical bone areas revealed irregular contours facing the intramedullary area (white arrows). None of the mentioned cortical alterations (microcracks, irregular contours) were identified in Sham bones. **b** Enlarged section of a 3R4F-exposed sample stained with Alcian blue (black square in (**a**) depicting hypertrophic chondrocytes in a microcrack area (black circle)), providing evidence of endochondral bone repair. Sham: *N* = 4; 3R4F: *N* = 2; CARRIER: *N* = 3; BASE: *N* = 3; TEST: *N* = 3

All groups were subjected to histological staining with H&E, Alcian blue, Masson trichrome stain, and Van Gieson stain (Fig. 5a) and a limited number of samples were investigated (2–4/group). H&E staining revealed microcracks in cortical areas in all treatment groups, depicting an interruption in cortical bone with dehiscence filled with cells. To further identify these cracks as real bone defects or artefacts, Alcian blue staining was performed, which revealed the presence of hypertrophic chondrocytes within these areas of cracked cortical bone, providing evidence for endochondral bone repair (Fig. 5a black arrows, Fig. 5b). This emphasizes the fact that these microcracks were bona fide microfractures. Also, irregular contours in cortical areas were depicted in treatment groups, however, neither in Sham (Fig. 5a white arrows). Along with the phenomenon of microcracks in cortical areas this might reflect ongoing remodeling and a reduction on bone stability; however, further investigations are needed to identify these mechanisms in detail.

## Discussion

In the present study, we investigated the effect of e-vapor aerosol exposure compared with CS exposure on bone integrity over a period of 6 months in ApoE^–/–^ mice. Additionally, we designed this study to assess the effect of the different e-vapor aerosol components (humectants, nicotine, and flavor mix) on bone tissue. Overall, our results demonstrated that, relative to sham treatment, neither cortical bone structure nor bone strength was compromised because of exposure to e-vapor aerosol or its individual components. In contrast, CS exposure was associated with detrimental effects on the architecture and biomechanical properties of bones.

ApoE^–/–^ mice were chosen for this study because this mouse model allows concomitant assessment of the cardiovascular and respiratory effects of CS exposure and potential electronic nicotine delivery systems (Lo Sasso et al. 2016; von Holt et al. 2009)—this assessment was further extended to bone-related endpoints in the current study.

Exposure characterization demonstrated the expected uptake of CS and e-vapor aerosol components across the experimental groups. Nicotine and cotinine reached systemic levels in blood and urine in all animals that were exposed to nicotine as a component of the inhalation formulation (3R4F, TEST, and BASE) but not in those without nicotine (Sham and CARRIER). The high plasma HbCO concentrations observed in the 3R4F CS group, but not in any of the other groups, confirmed efficient exposure, consistent with the smoke chemistry of this reference cigarette (Roemer et al. 2012). To put the nicotine dose in context of human exposure, we calculated the human equivalent dose—for inhalation exposure not considering other possible routes such as oral or dermal exposure—on the basis of body surface area by dividing the mouse dose by a factor of 12.3 (U.S. Department of Health and Human Services et al. 2005). Given a target concentration of 35.2 µg nicotine/L in the smoke and a 3 h daily exposure duration (assuming 25 g body weight, 0.03 L/min respiratory minute volume, and complete uptake), the estimated delivered dose in mice was 7.6 mg nicotine/(day x kg body weight), corresponding to 37.2 mg nicotine/day for a human weighing 60 kg or approximately 37.2 cigarettes per day. For example, the average daily nicotine inhalation dose of an e-vapor product user has been reported to be 36.7 mg among vape shop customers in Greece (Diamantopoulou et al. 2019) and 27.7 and 29.7 mg among Dutch and Belgian vape shop customers, respectively (Smets et al. 2019). Interestingly, these values are also comparable to the average nicotine dose of cigarette smokers, which was determined to be 37.6 mg (Benowitz and Jacob 1984). Of note, the nicotine plasma concentrations (> 100 ng/ml) observed in the current and previous ApoE^–/–^ studies (Phillips et al. 2019; Phillips et al. 2016b) are greater than those observed in human smokers and e-vapor product users, which are commonly below 50 ng/ml (Dawkins et al. 2016; Muranaka et al. 1988; St Helen et al. 2016).

Cigarette smoking is reported to have detrimental effects on the musculoskeletal system (Duthon et al. 2014; Adams et al. 2001; Mills et al. 2011; Scolaro et al. 2014). Furthermore, findings in different animal models support these clinical observations, including those demonstrating decreased callus formation during rat tibia fracture healing (El-Zawawy et al. 2006) and decreased bone mineral density due to conventional smoke inhalation in a mouse model (Ajiro et al. 2010). The results of our study are consistent with these findings, demonstrating that 3R4F CS exposure significantly reduces the total BV fraction, hydroxyapatite content, and mechanical properties of cortical bone, leading to a significant decrease in bone stiffness and ultimate strength.

Not much is known about the effect of e-vapor aerosols on bone. To our knowledge, our study is the first to investigate the effect of e-vapor aerosols on bone structure and strength in vivo. To date, studies have focused on in vitro models for assessing the effects of e-vapor liquids/aerosols on osteoblast differentiation. Otero et al. (Otero et al. 2019) showed that e-vapor liquid exposure diluted in medium induced osteotoxicity and increase the expression of collagen type 1—in a flavor-mix-dependent but nicotine-independent manner—in human osteoblast-like MG-63 and SaOs-2 cells. However, this study did not include CS exposure and thus does not support direct comparison of effect sizes between e-vapor liquid and CS (extracts). Shaito et al. demonstrated that CS exposure impairs the in vitro differentiation of mesenchymal stem cells towards the osteoblast linage (Shaito et al. 2017). In this study, qualitatively similar effects were observed for the tested e-vapor extracts, but with less pronounced effects.

In contrast to CS, the e-vapor aerosols tested in the present study did not demonstrate a detrimental influence on bone structure or strength in the ApoE^–/–^ mouse model. In particular, CS exposure led to a significant reduction in BV fraction and hydroxyapatite content as well as bone stiffness and ultimate strength in this mouse model. In contrast, e-vapor aerosol exposure did not have any significant effect on the biomechanical or structural properties of bone relative to sham treatment.

Crotty Alexander et al. (Crotty Alexander et al. 2018) reported increased fibrosis on a multi-organ level (kidneys, heart and liver) after e-vapor inhalation for 3 and 6 months in a mouse model. Szostak et al. (Szostak et al. 2020) did not reveal these findings for our model showing histopathological analysis of the mice’ hearts. However, neither for 3R4F nor for the e-vapor aerosol exposure a significant induction of fibrotic changes compared with Sham was identified. Our data are focusing on bone. Histological analysis did not reveal signs of fibrosis. However, further mechanistic analysis would be needed to determine changes on a molecular level.

The effect of nicotine on bone has been described above. Our previous data showed that, at concentrations observed in the serum of smokers, nicotine does not cause a decrease in osteogenic differentiation (Aspera-Werz et al. 2018). This supports our present data showing no evidence that bone structure and strength were affected by nicotine. Additionally, the findings of Otero et al. revealed no association between nicotine and osteoblast toxicity (Otero et al. 2019). This finding is in line with our results, as our in vivo data did not show an effect on bone structure and strength in the study groups with respect to nicotine exposure alone. Additionally, Skott et al. (Skott et al. 2006) showed that biomechanical bone strength in a fracture healing animal model was rather altered by tobacco extract plus nicotine exposure rather than by nicotine exposure only; in this study, tobacco extract was administered orally in drinking water.

Our histological analysis for visualizing bone morphology did not show any differences among the treatment groups. However, the findings of this method are purely descriptive and limited because of the small sample numbers. Further investigation is needed to identify the effects of smoking on i.e., collagen structure. Shaito et al. (Shaito et al. 2017) showed that CS and e-vapor aerosol extracts both caused a reduction in collagen-1 and Runx2 expression as well as alkaline phosphatase activity on mesenchymal stem cell differentiation towards the osteoblast lineage. This could reflect changes in the bone matrix. Thus, detailed research focusing on the effects of e-vapor aerosols on bone matrix quality is desirable.

Strength and limitations of the current study. Compared with other studies that investigated the effects of CS on bone in rodent models that exposed only for a period of 4–12 weeks (Ajiro et al. 2010; Akhter et al. 2005; El-Zawawy et al. 2006), the present study—with its 6-month time frame—allowed us to monitor the effects over a longer term. However, studies with an even longer exposure time may be considered. The current study focused on the apical endpoints of bone structure, strength, and morphology rather than on molecular changes.

We selected the ApoE^–/–^ mouse model for this inhalation toxicology study because this model is especially well suited for concomitant assessment of systemic, respiratory, and cardiovascular effects (Szostak et al. 2020). It has been demonstrated that ApoE also regulates mouse bone phenotypes (Schilling et al. 2005). ApoE-deficient osteoblasts show a decreased uptake of vitamin K containing lipoproteins. This leads to an incomplete carboxylation of osteocalcin, which inhibits bone formation (Ducy et al. 1996). Lack of ApoE seems to lead to increased bone formation (Schilling et al. 2005). This effect on bone phenotype must be taken into consideration when interpreting here the described data regarding the effect of e-vapor and CS exposure. However, similar as for the cardiovascular and respiratory endpoints, the predisposition of the ApoE^–/–^ model to bone-related changes might have facilitated more sensitive detection of the effects of CS exposure on bone. In this study, comparisons were made based on the concurrent air control with ApoE^–/–^ background across all groups. However, similar to our data, others have described changes in the biomechanical properties of bone in female C57BL (ApoE^–/–^) mice, demonstrating a significant reduction in bone stiffness, yield load, and stress in a side-stream smoke-exposure group relative to a non-smoking control (Akhter et al. 2005); as in the present study, there was no significant intergroup difference in baseline body weight or tibial bone length and weight.

This study assessed not only the full e-vapor liquid formulation (TEST), but also the CARRIER (humectants) and BASE (humectants and nicotine). None of these formulations showed a negative effect on bone integrity, including those containing nicotine and the flavor blend. A recent in vitro study suggested that some flavor blends have a (relatively) higher impact on osteoblast-like cell proliferation (Otero et al. 2019). While these results do not necessarily directly translate to the in vivo situation, this further emphasizes the relevance of careful toxicological evaluation of e-vapor aerosol formulations.

While nicotine was not associated with a negative effect on bone integrity, the current study did not further elucidate the exact mechanisms contributing to the effect of CS exposure on the bone. Considering the substantially higher levels of HPHCs in CS compared with the e-vapor aerosols (Szostak et al. 2020), a direct effect of CS components or their metabolites is possible. However, other systemic effects—as indicated by the effect of 3R4F CS on the full body weight trajectory (Szostak et al. 2020)—might also have contributed to the observed CS effects on bone integrity in the current study.

## Conclusions

In summary, to our knowledge, this study is the first to evaluate the effects of 6 months of inhalation of e-vapor aerosol and its various components relative to CS inhalation on bone in an ApoE^–/–^ mouse model. Our data revealed a significant decrease in cortical bone structure and strength due to CS inhalation, while inhalation of e-vapor aerosol and its individual components did not cause significant bone changes relative to Sham exposure.
